# Case Report: Epistaxis with electroconvulsive therapy: a report of 2 patients

**DOI:** 10.3389/fpsyt.2025.1443630

**Published:** 2025-06-20

**Authors:** Xin Yang, Shuping Fang, Yang Liu, Jin Li, Weihong Kuang

**Affiliations:** ^1^ Mental Health Center, West China Hospital, Sichuan University, Chengdu, Sichuan, China; ^2^ National Clinical Research Center for Geriatrics, West China Hospital, Sichuan University, Chengdu, Sichuan, China

**Keywords:** depression, electroconvulsive therapy, epistaxis, Little’s area, case report

## Abstract

Electroconvulsive therapy (ECT) is an effective treatment for various psychiatric disorders such as depression, bipolar disorder, and schizophrenia but is associated with certain side effects. Epistaxis following ECT appeared to be rare, with few reports in the medical literature. We reported two cases of epistaxis following ECT. In one case, we observed a sudden transient increase in blood pressure during ECT, followed by epistaxis. In the other case, the patient developed epistaxis after ECT, with subsequent ECT sessions leading to increasingly difficult-to-control nosebleeds. Nasal endoscopy revealed vascular engorgement in the Little’s area. Our cases suggested that sudden increases in blood pressure during ECT may trigger epistaxis. Repeated ECT sessions may exacerbate this issue, making epistaxis more challenging to manage. Clinicians must be aware of this potential adverse event and should reevaluate the efficacy and risks of ECT for patients who develop epistaxis.

## Introduction

Electroconvulsive therapy (ECT) has been in existence for over 80 years and is a medical procedure performed under general anesthesia, which induces seizures by applying electrical currents to the skull. It is effective in treating various psychiatric illnesses such as depression, bipolar disorder, and schizophrenia (1) and may also be a treatment option for neurological patients with mood disorders (2). ECT is known to rapidly alleviate depressive, psychotic, and catatonic symptoms, decrease suicidal ideation, and improve functional outcomes and quality of life (3, 4). Despite its clinical efficacy, ECT is associated with a range of side effects, primarily attributable to anesthesia, anticholinergic medications, muscle relaxants, electrical stimulation, or seizure activity (5). The estimated mortality rate associated with ECT is very low (2.1 deaths per 100,000 treatments), and serious adverse events related to ECT are rare, including cardiac arrhythmias with or without hemodynamic changes, respiratory distress, prolonged apnea, aspiration, and prolonged paralysis and seizures (6). Common self-limiting or symptomatic side effects include nausea, headache, muscle pain, increased blood pressure and acute cognitive adverse effects (5). Post-ECT epistaxis is rare, with scant documentation in medical literature. We report two unique cases of epistaxis following ECT. In one case, we observed a sudden transient increase in blood pressure during ECT, which was subsequently followed by epistaxis. In the other case, the patient developed epistaxis after ECT, and the subsequent ECT session resulted in more difficult-to-control epistaxis. Our case indicated that a sudden increase in blood pressure during ECT may trigger epistaxis. For patients who have already experienced epistaxis, the efficacy and risks of ECT should be re-evaluated.

## Case presentation

### Case 1

A 20-year-old female with a one-year history of depression and prior antidepressant treatment was admitted to the hospital due to a one-month relapse characterized by worsening low mood, feelings of worthlessness, suicidal ideation, and non-suicidal self-injury. She also reported experiencing auditory and visual hallucinations. Upon admission, her score on the 24-item Hamilton Depression Rating Scale (HAMD-24) was 35, indicating severe depression. She had previously been treated with sertraline, lamotrigine, and lorazepam, and was maintained on sertraline and lamotrigine prior to admission. The patient had no previous experience with electroconvulsive therapy (ECT). Upon psychiatric evaluation based on ICD-11 criteria (7), she was diagnosed with recurrent depressive disorder, current episode severe with psychotic symptoms, following comprehensive clinical interviews conducted by two experienced psychiatrists. The patient had no history of hypertension or diabetes and had not used anticoagulant medications.

At the time of admission, she was taking sertraline 100 mg/day and lamotrigine 25 mg twice daily. Because the patient had ongoing suicidal ideation, a clear suicide plan, and non-suicidal self-injurious behavior, and was rated as moderate risk on the Columbia-Suicide Severity Rating Scale (C-SSRS), along with having psychotic symptoms, ECT was considered a suitable treatment to quickly relieve symptoms and lower the immediate risk of suicide. Given the likely speed and efficacy of ECT relative to medication in MDD (8), treatment was initiated following a thorough clinical assessment, multidisciplinary team consultation, and informed consent from both the patient and her family. According to the American Psychiatric Association’s guideline on electroconvulsive therapy (9) and our institutional protocol, patients considered for ECT typically undergo a comprehensive pre-ECT medical evaluation, which includes physical examination, blood pressure monitoring, complete blood count, liver and kidney function tests, electrolytes, coagulation profile, electrocardiogram (ECG), brain magnetic resonance imaging (MRI), and chest computed tomography (CT). All test results were within normal limits. During ECT, anesthesia was induced with S-ketamine, and succinylcholine was used as a muscle relaxant, followed by the placement of bilateral electrodes to induce a seizure. The maximum charge was 504 mC, with 15% usage, a current of 800–900 mA, and a current duration of approximately 5 seconds. The seizure typically lasts approximately 20–40 seconds. During the fifth ECT session, we observed a sudden increase in blood pressure from 125/75 mmHg to 140/85 mmHg. After the seizure ceased, the blood pressure returned to 120/70 mm Hg. Following the ECT session, the patient experienced bilateral epistaxis, which stopped after applying pressure to the nasal wings. Further nasal endoscopy revealed mucosal ulceration in the right Little’s area, significant vasodilation in the left Little’s area and nasal septal deviation. To facilitate mucosal healing and minimize the risk of recurrent epistaxis, cod liver oil nasal drops, which are rich in vitamin A, were administered as a supportive topical treatment. After completing five ECT sessions, the patient showed marked clinical improvement. Her mood was significantly elevated and suicidal ideation had resolved, auditory and visual hallucinations were no longer reported, and there were no further episodes of non-suicidal self-injury. The HAMD-24 scores decreased from 35 to 16, indicating a significant reduction in symptom severity. Based on the notable clinical response and after a thorough multidisciplinary team evaluation of the patient’s improvement and the potential risks of continuing ECT, particularly concerns regarding nasal vascular dilation and mucosal fragility, it was concluded that her depressive symptoms had improved and the suicide risk was effectively managed. Therefore, the decision was made to discontinue further ECT sessions. The patient remained stable over the subsequent six days of inpatient observation, with no recurrence of epistaxis or psychiatric deterioration. She was discharged in good condition, and at the one-month follow-up, she continued to maintain emotional stability and functional recovery, with no return of either epistaxis or psychiatric symptoms.

### Case 2

An 18-year-old female with a history of depression lasting over a year was admitted to our hospital due to persistent low mood and suicidal ideation. On admission, she exhibited marked depressive symptoms, including depressed affect, psychomotor retardation, pronounced anhedonia, feelings of worthlessness, and both auditory and visual hallucinations. Her HAMD-24 score was 38, and the C-SSRS indicated a moderate suicide risk. Based on ICD-11 criteria (7), a diagnosis of a severe depressive episode with psychotic symptoms was confirmed by two senior psychiatrists following comprehensive clinical evaluation. The patient had no history of hypertension, diabetes, or use of anticoagulant medications.

Upon admission, pharmacological treatment was initiated with sertraline (50 mg/day) and aripiprazole (5 mg/day). Given the severity of her psychotic symptoms and suicidal ideation, and the need for a rapid and robust clinical response to reduce suicide risk, ECT was recommended. After obtaining informed consent, the patient underwent a comprehensive pre-ECT evaluation, including physical examination, blood pressure monitoring, ECG, complete blood count, liver and renal function tests, serum electrolytes, coagulation profile, brain MRI, and chest CT. All assessments were within normal limits. Similarly, this patient underwent induction anesthesia with S-ketamine and muscle relaxation with succinylcholine, followed by the application of bilateral electrodes to elicit a seizure, which lasted approximately 30–60 seconds. Following the third session, the patient experienced minor bilateral epistaxis, which was controlled by the anesthetist by applying pressure to the nasal wings. No acute increase in blood pressure was observed during ECT. Subsequent nasal endoscopy revealed marked vasodilation in the patient’s bilateral Little’s area, with the right side exhibiting more pronounced dilation. Cod liver oil nasal drops, which are rich in vitamin A, were administered as a supportive symptomatic treatment. Although her depressive symptoms improved modestly, including a reduction in psychomotor retardation and increased eye contact, the patient remained passively engaged with limited verbal output, and thus ECT was continued. After the fourth ECT session, she experienced a second episode of bilateral epistaxis, requiring nasal packing, adrenaline injection, and compression by the anesthesiology team for approximately 20 minutes. Repeat laboratory tests showed a hemoglobin drop from 128 g/L at admission to 108 g/L, while coagulation parameters remained within normal range. Given the decrease in hemoglobin levels and significant alleviation in suicidal ideation, as well as a notable reduction in depressive symptoms (HAMD-24 score decreased to 22), the ECT course was discontinued. Two weeks later, the patient’s mood had stabilized, with complete resolution of suicidal ideation and hallucinations, and no further episodes of epistaxis were observed. During this period, sertraline was gradually titrated to 150 mg/day and aripiprazole to 10 mg/day. The patient was subsequently discharged and scheduled for regular follow-up. At the one-month follow-up visit, she remained clinically stable, with no recurrence of epistaxis or psychotic symptoms.

## Discussion

Nasal bleeding is an infrequent adverse effect following ECT. A review of medical literature revealed that in a retrospective cohort study involving 32 patients treated with direct anticoagulants or warfarin and undergoing ECT, 1 case of nasal bleeding was reported (10). However, in the cases we present, neither patient had a history of anticoagulant use. This suggests that epistaxis can occur post-ECT even in patients not on anticoagulant therapy. Our cases highlight the need for heightened awareness among clinicians regarding the possibility of ECT-induced epistaxis, even in patients without anticoagulant therapy. Future research and case reports are critical in elucidating the incidence and mechanisms of epistaxis following ECT.

The occurrence of epistaxis following ECT has prompted us to re-evaluate potential underlying causes, including direct trauma during airway management, hemodynamic fluctuations during the procedure, anatomical anomalies of the nasal cavity, and the use of anticoagulants. In both of our reported cases, we observed vascular engorgement in the Little’s area, which is the source of approximately 80% to 90% of epistaxis cases (11). Previous studies have shown that during ECT, both sympathetic surge and the seizure itself can induce tachycardia, hypertension, and increased cardiac output, thereby causing hemodynamic alterations (5). Additionally, the anesthetic S-ketamine used during the ECT has sympathomimetic action, which can increase the risk of arterial hypertension (12). Both the seizure activity and the anesthetic may have a synergistic effect, collectively contributing to elevated arterial blood pressure. Our cases indicated that patients developed epistaxis post-ECT and did not experience recurrence after ECT was discontinued. A plausible hypothesis was that the hemodynamic alterations during ECT may affect the Little’s area, a convergence zone of multiple blood vessels, leading to increased congestion and causing the fragile small blood vessels to rupture and bleed. Further research is needed to validate this hypothesis. Moreover, our second case suggested that epistaxis can become increasingly difficult to control with repeated ECT sessions. After the third ECT session, the patient’s epistaxis could be managed with pressure alone, but following the fourth session, hemostasis required pressure, nasal packing, and the use of vasoconstrictors. Therefore, for patients experiencing epistaxis, it is necessary to reassess the potential benefits and risks of continuing ECT. Conservatively, temporarily halting ECT might be a prudent decision.

Additional factors need to be considered when assessing the risk of epistaxis in ECT patients. For example, the role of systemic and localized inflammation in the nasal mucosa, which might be exacerbated by repeated ECT, could contribute to increased fragility of nasal blood vessels. The potential impact of comorbid conditions such as hypertension, which may predispose patients to vascular rupture under stress, should also be evaluated. The anatomical characteristics of the nasal cavity, including any pre-existing conditions like a deviated septum or nasal polyps, could predispose patients to trauma during intubation or mask ventilation. In our cases, performing detailed nasal endoscopic evaluations post-epistaxis was crucial in ruling out such contributing factors. Performing nasal examinations before ECT, carefully managing the airway to minimize trauma, closely monitoring blood pressure and heart rate during the procedure, and using anesthetics with low sympathomimetic action may all reduce the risk of epistaxis.

## Conclusion

In conclusion, while ECT remains a highly effective treatment for various psychiatric disorders, awareness and management of rare adverse effects like epistaxis are vital. Our cases highlight the potential risk of epistaxis following ECT, even in patients not on anticoagulant therapy. The hemodynamic changes induced by ECT, along with the sympathomimetic effects of the anesthetic S-ketamine, may contribute to increased arterial blood pressure and subsequent nasal bleeding. Particularly, repeated ECT sessions may exacerbate this issue, making it increasingly difficult to control epistaxis. Therefore, it is essential for clinicians to be aware of this potential complication and consider a thorough nasal examination prior to ECT, meticulous airway management to minimize trauma, and the use of anesthetics with lower sympathomimetic activity. For patients who experience epistaxis, a reevaluation of the risks and benefits of continuing ECT is warranted. Further research is needed to better understand the incidence and mechanisms of post-ECT epistaxis, ultimately improving patient care protocols in this context.

## Data Availability

The original contributions presented in the study are included in the article/**Supplementary Material**. Further inquiries can be directed to the corresponding author.

## References

[1] Kritzer MD Peterchev AV Camprodon JA. Electroconvulsive therapy: mechanisms of action, clinical considerations, and future directions. Harvard Rev Psychiatry. (2023) 31:101–13. doi: 10.1097/HRP.0000000000000365 PMC10198476 37171471

[2] Elefante C Brancati GE Tripodi B Torrigiani S Lattanzi L Medda P. Catatonia in two women with Parkinson’s Disease treated with electroconvulsive therapy. Explor Neuroprotective Ther. (2022) 2:256–63. doi: 10.37349/ent

[3] Tor PC Tan XW Martin D Loo C. Comparative outcomes in electroconvulsive therapy (ECT): A naturalistic comparison between outcomes in psychosis, mania, depression, psychotic depression and catatonia. Eur neuropsychopharmacology: J Eur Coll Neuropsychopharmacol. (2021) 51:43–54. doi: 10.1016/j.euroneuro.2021.04.023 34034099

[4] Sienaert P Brus O Lambrichts S Lundberg J Nordanskog P Obbels J. Suicidal ideation and ECT, ECT and suicidal ideation: A register study. Acta psychiatrica Scandinavica. (2022) 146:74–84. doi: 10.1111/acps.v146.1 35279825 PMC9313798

[5] Andrade C Arumugham SS Thirthalli J. Adverse effects of electroconvulsive therapy. Psychiatr Clinics North America. (2016) 39:513–30. doi: 10.1016/j.psc.2016.04.004 27514303

[6] Espinoza RT Kellner CH. Electroconvulsive therapy. New Engl J medicine. (2022) 386:667–72. doi: 10.1056/NEJMra2034954 35172057

[7] World Health Organization. International classification of diseases 11th revision . Available online at: https://icd.who.int/en (Accessed April 15, 2024).

[8] UK ECT Review Group. Efficacy and safety of electroconvulsive therapy in depressive disorders: a systematic review and meta-analysis. Lancet. (2003) 361:799–808. doi: 10.1016/S0140-6736(03)12705-5 12642045

[9] APA Committee on Electroconvulsive Therapy. The practice of electroconvulsive therapy: recommendations for treatment, training, and privileging. 2nd Ed. Washington, DC: American Psychiatric Association (2001).

[10] Centanni NR Craig WY Whitesell DL Zemrak WR Nichols SD. Safety of ECT in patients receiving an oral anticoagulant. Ment Health clinician. (2021) 11:254–8. doi: 10.9740/mhc.2021.07.254 PMC8287866 34316422

[11] Seikaly H. Epistaxis. New Engl J medicine. (2021) 384:944–51. doi: 10.1056/NEJMcp2019344 33704939

[12] Zavorotnyy M Kluge I Ahrens K Wohltmann T Köhnlein B Dietsche P. S -ketamine compared to etomidate during electroconvulsive therapy in major depression. Eur Arch Psychiatry Clin Neurosci. (2017) 267:803–13. doi: 10.1007/s00406-017-0800-3 28424861

