# Supplementary material for: Case Report: Epistaxis with electroconvulsive therapy: a report of 2 patients
Source: Front Psychiatry. 2025 Jun 20;16:1443630. doi: 10.3389/fpsyt.2025.1443630 (PMC12226574; doi:10.3389/fpsyt.2025.1443630)
Supplement: Supplementary file 1 [file DataSheet1.docx]

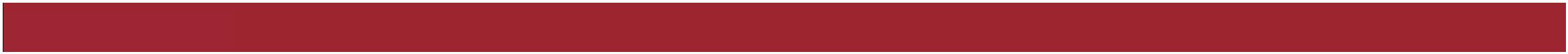

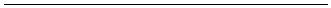

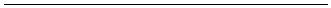

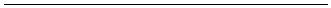

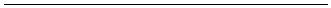

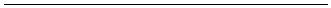

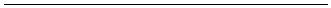

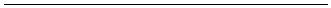

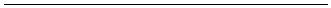

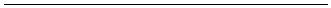

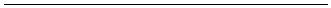

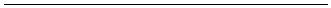

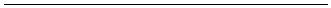

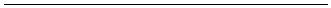

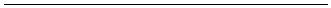

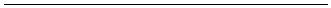

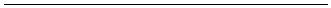

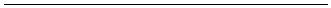

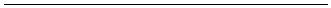

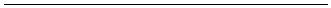

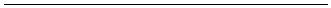

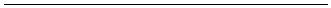

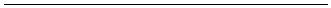

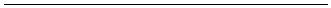

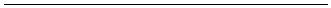

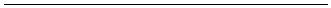

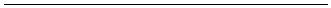

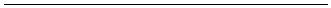

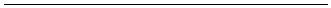

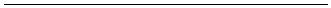

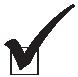

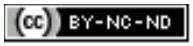

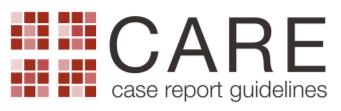
CARE Checklist of information to include when writing a case report

Topic Item Checklist item description Reported on Line

Title 1 The diagnosis or intervention of primary focus followed by the words “case report” . . . . . . . . . . . . . . . . . . . . . . . . . . √

Key Words 2 2 to 5 key words that identify diagnoses or interventions in this case report, including "case report" . . . √

|  | Abstract  (no references) |  | 3a Introduction: What is unique about this case and what does it add to the scientific literature? . . . . . . . . . . . . . . . .  3b Main symptoms and/or important clinical findings . . . . . . . . . . . . . . . . . . . . . . . . . . . . . . . . . . . . . . . . . . . . . . . . . . . . . . .  3c The main diagnoses, therapeutic interventions, and outcomes . . . . . . . . . . . . . . . . . . . . . . . . . . . . . . . . . . . . . . . . . . . |
| --- | --- | --- | --- |

√

√

√

3d Conclusion—What is the main “take-away” lesson(s) from this case? . . . . . . . . . . . . . . . . . . . . . . . . . . . . . . . . . . . . . √

Introduction 4 One or two paragraphs summarizing why this case is unique (may include references) . . . . . . . . . . . . √

Patient Information 5a De-identified patient specific information. . . . . . . . . . . . . . . . . . . . . . . . . . . . . . . . . . . . . . . . . . . . . . . . . . . . √

5b Primary concerns and symptoms of the patient. . . . . . . . . . . . . . . . . . . . . . . . . . . . . . . . . . . . . . . . . . . . . . . . . . . . . √

5c Medical, family, and psycho-social history including relevant genetic information . . . . . . . . . . . . . . . . . √

5d Relevant past interventions with outcomes . . . . . . . . . . . . . . . . . . . . . . . . . . . . . . . . . . . . . . . . . . . . . . . . . . . . . . . . √

Clinical Findings 6 Describe significant physical examination (PE) and important clinical findings. . . . . . . . . . . . . . . . . . . . . . . √

Timeline

7 Historical and current information from this episode of care organized as a timeline . . . . . . . . . . . . . . . √

| Diagnostic  Assessment |  | 8a Diagnostic testing (such as PE, laboratory testing, imaging, surveys). . . . . . . . . . . . . . . . . . . . . . √  8b Diagnostic challenges (such as access to testing, financial, or cultural) . . . . . . . . . . . . . . . . . . . . . . . . |
| --- | --- | --- |

N/A

8c Diagnosis (including other diagnoses considered) . . . . . . . . . . . . . . . . . . . . . . . . . . . . . . . . . . . . . . . . . . . . . . . . √

8d Prognosis (such as staging in oncology) where applicable . . . . . . . . . . . . . . . . . . . . . . . . . . . . . . . . . . . . . . . . N/A

| Therapeutic  Intervention | | |  | 9a Types of therapeutic intervention (such as pharmacologic, surgical, preventive, self-care) . . . . . . . . . . . . . . . .  9b Administration of therapeutic intervention (such as dosage, strength, duration) . . . . . . . . . . . . . . . . . . . . . . . . . . . . .  9c Changes in therapeutic intervention (with rationale) . . . . . . . . . . . . . . . . . . . . . . . . . . . . . . . . . . . . . . . . . . . . . . . . . . . . |  |
| --- | --- | --- | --- | --- | --- |
| Follow-up and  Outcomes |  | 10a Clinician and patient-assessed outcomes (if available) . . . . . . . . . . . . . . . . . . . . . . .. . . . . . . . . . . . . . . . . . . . . . . . . . . .  10b Important follow-up diagnostic and other test results . . . . . . . . . . . . . . . . . . . . . . . . . . . . . . . . . . . . . . . . . . . . . . . . . . . . | | | |

√

√

√


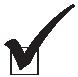
√

√
10c Intervention adherence and tolerability (How was this assessed?) . . . . . . . . . . . . . . . . . . . . . . . . . . . . . . . . . . . . . . . . . √

10d Adverse and unanticipated events . . . . . . . . . . . . . . . . . . . . . . . . . . . . . . . . . . . . . . . . . . . . . . . . . . . . . . . . . . . . . . . . . . .
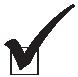
 √

Discussion 11a A scientific discussion of the strengths AND limitations associated with this case report . . . . . . . . . . . . . . . . . . . . . . . √

11b Discussion of the relevant medical literature with references. . . . . . . . . . . . . . . . . . . . . . . . . . . . . . . . . . . . . . . . . . √

11c The scientific rationale for any conclusions (including assessment of possible causes) . . . . . . . . . . . . . . . . . . . . . . . . √

11d The primary “take-away” lessons of this case report (without references) in a one paragraph conclusion . . . . . . . √

Patient Perspective 12 The patient should share their perspective in one to two paragraphs on the treatment(s) they received . . . . N/A


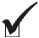

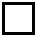

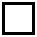
Informed Consent 13 Did the patient give informed consent? Please provide if requested . . . . . . . . . . . . . . . . . . . . . . . . . . . . . . . . . . . . . . Yes No
